# Effects of Replacing Soybean Meal with Sunflower Meal or Fermented Sunflower Meal on the Growth Performance, Intestinal Microbiota, and Intestinal Health of Tilapia (GIFT, *Oreochromis niloticus*)

**DOI:** 10.1155/2024/9366952

**Published:** 2024-06-20

**Authors:** Huajing Huang, Yu Liu, Hang Zhou, Xiangqin Lin, Xuehan Wang, Wen Jiang, Lu Zhang, Haifeng Mi, Junming Deng

**Affiliations:** ^1^ College of Fisheries Guangdong Ocean University, Zhanjiang 524088, China; ^2^ Aquatic Animals Precision Nutrition and High-Efficiency Feed Engineering Research Centre of Guangdong Province Guangdong Ocean University, Zhanjiang 524088, China; ^3^ Tongwei Agricultural Development Co. Ltd., Chengdu 610093, China

## Abstract

A 9-week feeding trial was conducted to evaluate the effects of replacing soybean meal (SBM) with sunflower meal (SM) or fermented sunflower meal (FSM) on the growth performance, intestinal microbiota, and intestinal health of genetically improved farmed tilapia (*Oreochromis niloticus*) (initial weight 6.55 ± 0.01 g). Eleven isonitrogenous and isolipidic experimental diets were formulated by replacing 0%, 20%, 40%, 60%, 80%, and 100% of dietary SBM with SM or FSM. The results showed that the replacement of more than 40% of SBM with SM decreased the weight gain and special growth rate of tilapia, while the complete replacement of SBM with FSM did not affect the growth performance of tilapia. From transmission electron microscopy analyses, it was shown that high levels of both SM and FSM substitution resulted in damage to the intestinal epithelium of tilapia. Replaced of 20% SBM with SM upregulated intestinal tight junction (*zo-1*, *claudin*, *occludin*) and anti-inflammatory (*tgf-β1*, *tgf-β2*) gene expression and downregulated pro-inflammatory gene expression (*tnf-α*, *il-1β*, *il-6*, *il-8*). However, the expression of tight junction, anti-inflammatory, and pro-inflammatory genes showed opposite trends when SBM was substituted by SM at high levels. FSM completely replaces SBM and downregulates the expression of tight junction genes (*claudin*, *occludin*), replacement of more than 20% of SBM with FSM downregulated pro-inflammatory (*tnf-α*, *il-1β*, *il-8*) gene expression, whereas substitution of less than 80% increased the expression of anti-inflammatory genes (*tgf-β1*). The 100% FSM group exhibited a decreased abundance of Fusobacteriota and an increased abundance of Actinobacteriota compared to the control and 100% SM groups. In summary, our data confirm that replacing more than 40% of SBM with SM induces gut inflammation, damages gut health, and decreases growth performance, whereas FSM replacement of SBM did not negatively affect tilapia growth and health, it also did not have a significant ameliorative effect, with some parameters negatively affected at high replacement levels. Therefore, FSM replacement of SBM levels above 80% is not recommended.

## 1. Introduction

In recent years, China's aquaculture industry has undergone vigorous development, and the demand for feed is increasing. This has resulted in continuous increases in feed grain use. Nevertheless, China has a large population and, thus, a large demand for food. Food competition between humans and farmed animals is a growing problem in China [1]. In particular, there is fierce competition for soybeans and the byproducts of soybean processing (soybean meal (SBM) and soybean oil), which are commonly used as animal feed ingredients but are also popular foods for humans. Therefore, to protect national food security, there has been intense interest in the development of nongrain protein ingredients to replace SBM in animal feed. Of note, the price of nongrain proteins is relatively low, and the use of these alternative proteins will effectively reduce feed costs and contribute to the development of aquaculture.

At present, the development of agricultural byproduct waste is recognized as the core approach to reducing and replacing SBM in feed. Such byproducts include rapeseed meal, palm kernel meal, and sunflower meal (SM). Among them, SM has a high nutritional value and large yield, offering good application prospects. Depending on the degree of dehulling and the oil extraction process, the protein content of SM ranges from 29% to 45% [2]. Compared to SBM, SM is inexpensive, is in stable supply, has less antinutritional factors except tannin and phytic acid, is low in sulfur-containing amino acids, and has a good balance of other amino acids [3, 4, 5]. In fish feed, it has been added to the diets of Nile tilapia (5.2%) (*Oreochromis niloticus*) [6], Turbot (12.9%) (*Scophthal musmaximus* L) [7], grass carp (19.06%) (*Ctenopharyngodon idellus*) [8], common carp (21%) (*Cyprinus carpio*) [4], and tilapia (21.58%) (*Tilapia rendelli*) [9] with good utilization effects. Current research shows that the addition of low doses of SM to feed does not affect the growth performance of aquatic animals (5.2%–21.58%), but excessive levels inhibit growth performance due to the high contents of fiber (18%–23%) and lignin [10].

In order to improve the utilization of SM, methods to improve the utilization of plant protein sources have been studied, including bacteria and enzyme synergistic fermentation, microbial fermentation, heat treatment, enzymatic hydrolysis, and expansion treatment. In particular, the synergistic fermentation of bacteria and enzymes has exhibited superior results, with better fermentation results in shorter fermentation times [11]. Bacterial and enzyme synergies can increase the contents of nutrients, including macromolecular proteins, amino acids, mineral elements, and specific functional small peptides. At the same time, this approach can improve the quality of the feed, enhance its palatability and facilitate the digestion and absorption of nutrients by the animal [12]. Fermentation reduces or completely eliminates antinutritional factors [13] and improves animal growth performance and immunity, promoting intestinal health. Currently, bacterial or enzyme synergistic fermentation techniques have been applied to various raw materials [13, 14]. Existing studies have found that only single fermentation and enzymatic hydrolysis methods are currently used on SM [6, 8]. However, there are no relevant studies on the effects of the fermentation of SM with bacteria and enzymes on aquatic animals.

Genetically improved farmed tilapia (GIFT, *O. niloticus*) is widely farmed due to its delicious taste, high disease resistance, and fast rate of growth [15, 16]. To date, a substantial amount of research has examined the nutrition of this species, but there remains a lack of research on SM and fermented sunflower meal (FSM). Therefore, this study comprehensively evaluated the effects of replacing SBM with SM or FSM on the growth performance, intestinal microbiota, and intestinal health of GIFT tilapia. These results will offer a theoretical basis for more effective use of SM and FSM in aquafeed.

## 2. Materials and Methods

### 2.1. Animal Ethical Statement

The Animal Research and Ethics Committee of Guangdong Ocean University (GDOUIACUC-2021-A0207) approved this experiment, and all experimental procedures were conducted in accordance with the Guidance of the Care and Use of Laboratory Animals in China (GB/T 35892-2018).

### 2.2. Experimental Diets

SM and FSM were provided by Tongwei Group (Tongwei Co., Ltd., Chengdu, China). The specific fermentation process, the preparation of bacterial and enzyme solution, was carried out with strict reference to the method provided by Vland Biotech (Qingdao, China). Before preparing FSM, One tonne of SM was added to 400 L of water and 10 kg of brown sugar. Then, 50 L of bacterial solution (yeast, lactic acid bacteria, and bacillus) and 1 L of enzyme solution (protease, nonstarch polysaccharide, and cellulase enzyme) were added, and the materials were mixed well. Next, the materials were placed into plastic bags and compacted and sealed. Finally, After 4 days, take a sample of the fermented feed and add the material to distilled water at a ratio of 1 : 5. When the pH of the solution measured below 5.0, the fermentation was terminated. The nutrient levels of SM and FSM are presented in Table 1.

Eleven isonitrogenous (crude protein 31%) and isolipidic (crude lipid 7%) experimental diets were formulated. SM or FSM replaced 0, 20%, 40%, 60%, 80%, and 100% of SBM in the basic diet (Table 2). All materials were crushed, put through a 40-mesh sieve, precisely weighed using the feed formula, thoroughly combined, and mixed well; soybean lecithin and soybean oil were added and mixed again. Finally, add the right amount of water and mix thoroughly again. Using a twin-screw mixer, they were extruded into pellets of 2.00 mm (F-26II, South China University of Technology, Guangzhou, China). They were then dried naturally and stored at −20°C in a refrigerator. The molecular weight distributions of the proteins are shown in Figure S1.

### 2.3. Feeding Experiment

Experimental tilapia obtained from the Lianjiang branch of Hainan Baolu Aquatic Technology Co., Ltd. (Guangdong, China) were fed a commercial diet (Jiakang feed, Xiamen, China) for 14 days to acclimatize them to the experimental conditions. Then, fish of a similar size (6.55 ± 0.01 g) were randomly assigned into 33 cages (1.2 m × 0.8 m × 1.0 m) with 35 juveniles per cage (11 experimental treatment groups, 3 replicates in each group). The cages were placed outdoors in two concrete ponds adjacent to each other (5.8 m × 5.4 m × 2.1 m). The water source was filtered groundwater. During the 9-week feeding period, the fish were fed twice daily (08:00 and 17:00) to satiation and were kept under a natural photoperiod and continuous aeration. The breeding water temperature ranged from 25 to 30°C, and the dissolved oxygen was >6.0 mg/L.

### 2.4. Sample Collection

Digestibility tests were conducted using Y_2_O_3_ (99.9%, Sinopharm Chemical Reagent Co., Ltd., Shanghai, China) as an indicator in the test feeds. Fecal samples were collected and preserved with reference to previous studies [1] and the methods of Liu et al. [17]. At the conclusion of the experimental feeding period, all fish were fasted for a day, and the number and total weight of the fish in each net were recorded to calculate the growth index. The fish were then anesthetized with a solution of eugenol (1 : 12,000) (Shanghai Reagent Corporation, Shanghai, China). Sampling strategies, sample preparation, and preservation methods were carried out as described in previous studies [1].

### 2.5. Chemical Composition Analysis

The chemical compositions of the feed ingredients, experimental diets, and feces were determined on the AOAC [18] standard methods. Dry matter, crude protein, crude lipid, crude ash, amino acids, and total energy content of the samples were performed according to the methods described in previous studies [1]. The protein macromolecules were determined by sodium dodecyl sulfate-polyacrylamide gel electrophoresis (SDS–PAGE) with reference to Bian's method [19].

### 2.6. Analysis of Biochemical Indices

The foregut amylase and lipase activities were measured by using commercial kits (Nanjing Jiancheng Bioengineering Institute, Nanjing, China). The serum endothelin content, diamine oxidase activity, and the foregut trypsin activity were measured by using commercial kits, according to the manufacturer's instructions (Shanghai Enzyme-Linked Biotechnology Co., Ltd., Shanghai, China).

### 2.7. Histological Observation

The hindguts were placed in a 4% paraformaldehyde solution, dehydrated with different gradients of ethanol, washed in toluene, and embedded in paraffin wax to obtain solid wax blocks. The solid wax blocks were then cut into 5-*μ*m-thick slices using a pathology slicer (RM2016, Leuchtenka, Germany). Then, hematoxylin-eosin staining was performed, encapsulated, and made into sections. The intestinal morphological structure was observed and photographed using the method of Huang et al. [1]. The hindgut tissue was preserved in a 2.5% glutaraldehyde fixation solution and stored away from light; sections for the intestinal transmission electron microscope (TEM) were created using Huang's method [20].

### 2.8. Gene Expression Analysis

RNA extraction, reverse transcription, and quantitative real-time PCR reactions were performed according to the methods described in previous studies [1]. Served *β*-actin as the internal reference gene. The relative expression levels of genes were determined using the ^2−*ΔΔ*CT^ technique [21]. The sequences of gene primers are shown in Table 3.

### 2.9. Intestinal Microbiota Analysis

The hindgut samples were sent to Guangzhou Genede novo Biotechnology Co., Ltd. (Guangzhou, China) for processing. After quality testing using an ultraviolet (UV)-spectro-photometer (Thermo Fisher Scientific, USA), bacterial DNA was extracted using the HiPure Soil DNA kit (Magen, China). The details of the procedure were referred to the study of Ye et al. [22]. The V3–V4 region of the bacterial 16S rRNA gene fragment was amplified using universal primers (341F: 5′-CCTACGGGGNGGCWGCAG-3′;806R: 5′-GGACTACHVGGGGTATCTAAT-3′).

### 2.10. Statistical Analysis

After confirming the normal distribution and homogeneity of variance for all data. First, a one-way ANOVA was conducted for different levels of substitution for each of the two feedstocks to assess the effect of the level of substitution under the same feedstock using Duncan's test of significance. Then, two-way ANOVA was performed to assess the effect of ingredient and substitution levels. The mean of all the substitution levels under both the ingredients were taken separately, and an independent samples *t*-test was conducted to assess the effect of the ingredient. The mean values of both the ingredients under the same level of substitution were taken and a one-way ANOVA was conducted to assess the impact of the level of substitution by Duncan's test of significance. Differences were considered significant at the *P* < 0.05 level. Statistical calculations were performed using SPSS 25.0 (SPSS Inc., Chicago, IL, USA).

## 3. Results

### 3.1. Growth Performance

Ingredients or replacement level significantly affected FBW, weight gain (WG), FI, special growth rate (SGR), FCR, and PER (*P* < 0.05, Table 4), but there was no significant interaction between ingredients and replacement level (*P* > 0.05). There was no significant increase in FBW, WG, and SGR when the replacement level was increased from 40% to 100% (*P* > 0.05), whereas there was no significant increase in FI and FCR when the level of substitution was increased from 20% to 80% (*P* > 0.05). FBW, WG, FI, SGR, and PER were significantly higher in fish from the FSM group than in the SM group (*P* < 0.05). FBW, WG, and SGR were significantly lower at replacement levels above 40% when the ingredients were SM (*P* < 0.05).

### 3.2. Digestive Enzyme Activity

Ingredients or replacement level significantly decreased amylase and trypsin activities (*P* < 0.05, Table 5), but there was no significant interaction between ingredients and replacement level (*P* > 0.05). There was no significant increase in amylase, lipase, and trypsin activities when the replacement level was increased from 60% to 100% (*P* < 0.05). Amylase and lipase reached their maximum at 20% replacement level and trypsin reached its maximum at 40% replacement level when ingredients were SM. When ingredients were FSM, trypsin activity was significantly reduced at substitution levels above 40% (*P* < 0.05).

### 3.3. Apparent Digestibility

Ingredients or replacement level and their interaction significantly affected dry matter and energy (*P* < 0.05, Table 6). Ingredients and interactions had significant effects on crude protein, crude lipid, and crude ash (*P* < 0.05). Dry matter, energy, crude protein, crude lipid, and crude ash were significantly higher in fish from the FSM group than in the SM group (*P* < 0.05). When ingredients were SM, dry matter, energy, crude protein, crude lipid, and crude ash gradually decreased with increasing replacement level, while FSM showed the opposite trend.

### 3.4. Intestinal Mucosal Barrier Status

**Figure 1:**
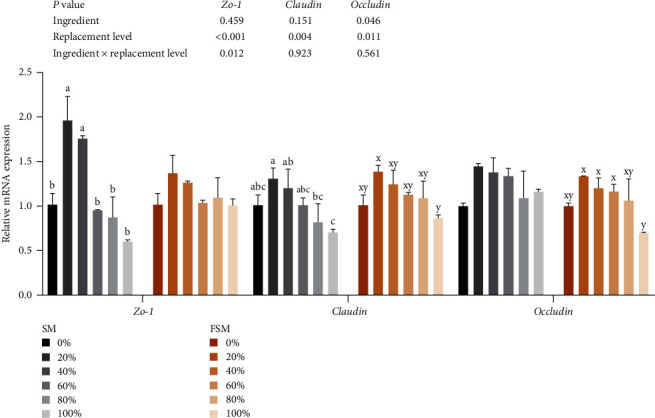
Effect of replacing soybean meal (SBM) with sunflower meal (SM) and fermented sunflower meal (FSM) on hindgut tight junction-related gene expression of tilapia. Data are expressed as mean ± SEM (*n* = 3). ^a,b,c^ Means with different superscript letters in the same column are significantly different (*P* < 0.05) among treatments with SM; ^x,y^ means with different superscript letters in the same column are significantly different (*P* < 0.05) among treatments with FSM. *Zo-1*: zona occludens protein 1.

Ingredients and replacement level and their interaction had significant effects on diamine oxidase and endothelin (*P* < 0.05, Table 7). Diamine oxidase activity was significantly reduced at substitution levels of 20%–80% (*P* < 0.05). Diamine oxidase activity and endothelin content were minimized at 20% replacement level when ingredients were SM or FSM (*P* < 0.05). Replacement level significantly affected *zo-1*, *claudin*, and *occludin* mRNA levels (*P* < 0.05, Figure 1). There was a significant interaction effect between ingredients and replacement level on *zo-1* mRNA levels (*P* < 0.05). As the SM and FSM replacement levels increased, *zo-1*, *claudin*, and *occludin* mRNA levels tended to increase and then decrease, with maximum values at 20%.

### 3.5. Intestinal Morphology

**Figure 2:**
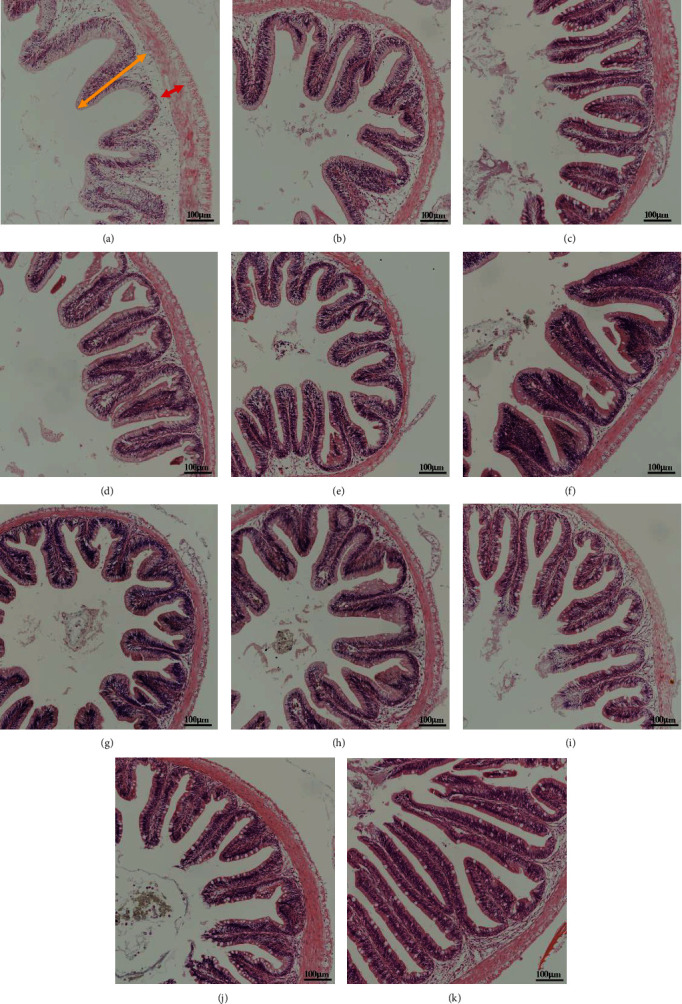
Effect of replacing soybean meal (SBM) with sunflower meal (SM) and fermented sunflower meal (FSM) on hindgut histology of tilapia: (a) control; (b) 20% SM; (c) 40% SM; (d) 60% SM; (e) 80% SM; (f) 100% SM; (g) 20% FSM; (h) 40% FSM; (i) 60% FSM; (j) 80% FSM; (k) 100% FSM. Red arrows: muscular thickness (*μ*m); yellow arrows: fold height (*μ*m). The magnification was ×200, and the minimum scale (lower right) was 100 *µ*m.

As shown in Table 8 and Figure 2, when the ingredients were SM, the trend of increase and then decrease of fold height and muscular thickness was observed with the increase of replacement level, and the maximal value of fold height was obtained at 40% of replacement level, while the maximal value of muscular thickness was obtained at 20% of replacement level. When the ingredients were FSM, the maximum value of fold height was obtained at 80% of the replacement level, and there was a significant interaction effect of ingredients and replacement level on fold height and muscular thickness (*P* < 0.05).

### 3.6. TEM of Intestinal Mucosal Cell

**Figure 3:**
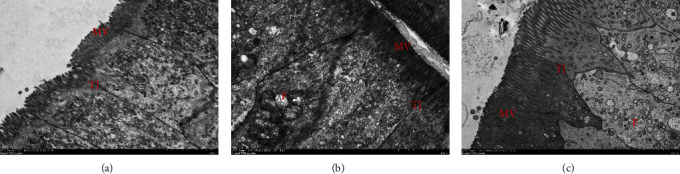
Transmission electron microscope of the intestine of tilapia: (a) control; (b) 100% SM; (c) 100% FSM; MV: microvilli; TJ: tight junction; F: mitochondrial swelling (magnification 7,000).

The TEM analysis showed a dense microvillus linkage structure between the mucosal cells in control, 100% SM, and 100% FSM groups (Figure 3); the structure was tight, and there were no gaps between the cells. However, damage to mitochondria was observed in both the 100% SM and 100% FSM groups.

### 3.7. Intestinal Immune Status

**Figure 4:**
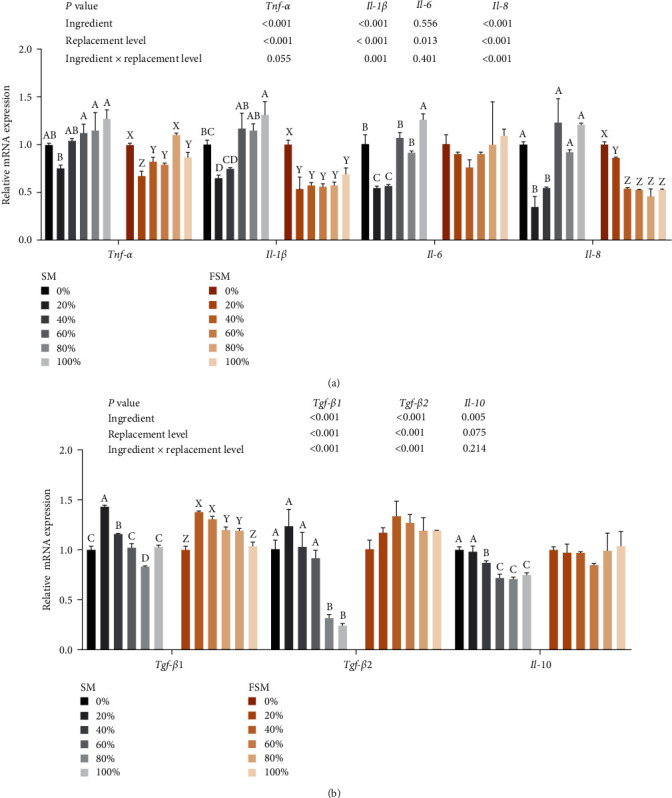
Effect of replacing soybean meal (SBM) with sunflower meal (SM) and fermented sunflower meal (FSM) on hindgut inflammation-related gene expression of tilapia. Data are expressed as mean ± SEM (*n* = 3). ^A,B,C,D^ Means with different superscript letters in the same column are significantly different (*P* < 0.05) among treatments with SM; ^X,Y,Z^ means with different superscript letters in the same column are significantly different (*P* < 0.05) among treatments with FSM. *Tnf-α*: tumor necrosis factor-alpha; *Il-1β*: interleukin-1 beta; *Il-6*: interleukin- 6; *Il-8*: interleukin-8; *Tgf-β1*: transforming growth factor *β*1; *Tgf-β2*: transforming growth factor beta 2; *Il-10*: interleukin-10. (a) pro-inflammatory factor-related gene expression and (b) anti-inflammatory factor factor-related gene expression.

Ingredients or replacement level and their interaction significantly affected *il-1β*, *il-8*, *tgf-β1*, and *tgf-β2* (*P* < 0.05, Figures 4(a) and 4(b). When ingredients were SM, *tnf-α*, *il-1β*, *il-6*, and *il-8*, mRNA levels showed a decreasing and then increasing trend with increasing replacement levels, and a minimum value was achieved at 20%, while the opposite trend was observed for *tgf-β1* mRNA levels. When the ingredients were FSM, *tnf-α*, *il-1β*, *and il-8*, mRNA levels were significantly reduced with increasing replacement levels (*P* < 0.05).

### 3.8. Intestinal Microbiota

#### 3.8.1. *α*-Diversity Analysis

When ingredients were SM or FSM, Shannon, Simpson, Chao1, and Ace tended to increase as the level of substitution increased, with a maximum value achieved at 100% (*P* < 0.05, Table 9). There was no significant interaction effect of ingredients and replacement level on Shannon, Simpson, Chao1, and Ace (*P* > 0.05).

#### 3.8.2. Microbial Composition

**Figure 5:**
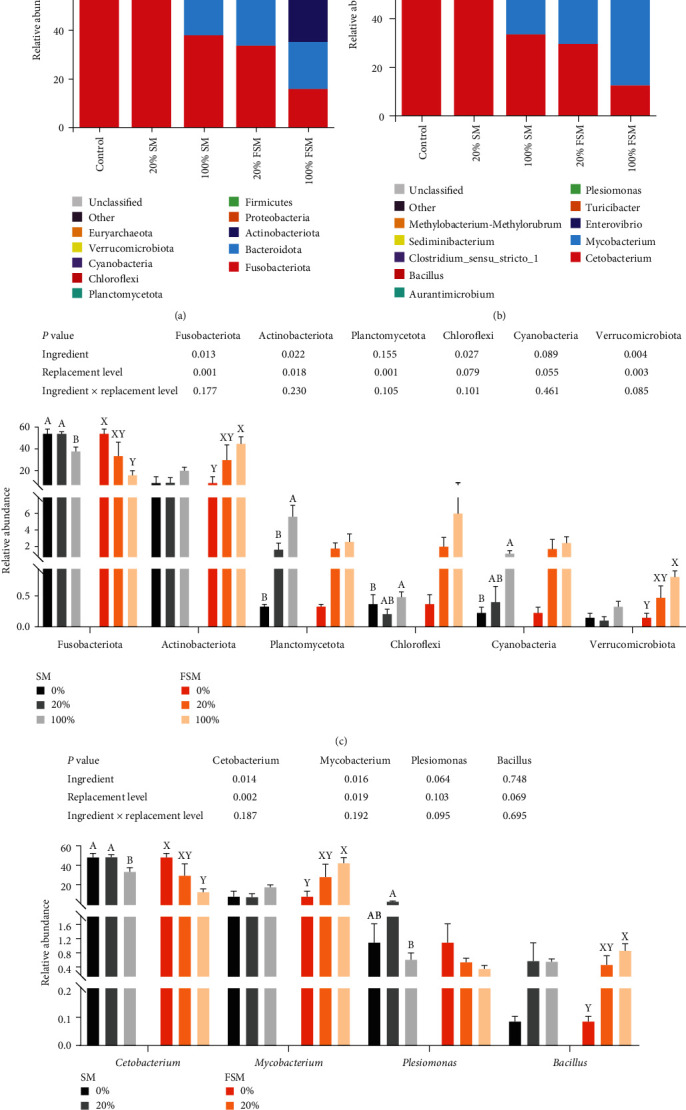
Effect of replacing soybean meal (SBM) with sunflower meal (SM) and fermented sunflower meal (FSM) on intestinal flora of tilapia: (a) phylum level; (b) genus level; (c) relative abundance is significantly different at the phylum level (top 10); (d) relative abundance is significantly different at the genus level (top 10). Data are expressed as mean ± SEM (*n* = 4). ^A,B^ Means with different superscript letters in the same column are significantly different (*P* < 0.05) among treatments with SM; ^X,Y^ means with different superscript letters in the same column are significantly different (*P* < 0.05) among treatments with FSM.

As shown in Figure 5(a), the dominant bacteria in the tilapia intestinal microbiota were Fusobacteriota, Bacteroidota, Actinobacteriota, and Proteobacteria at the phylum level. Ingredients significantly affected Fusobacteriota, Actinobacteriota, and Chloroflexi abundance (*P* < 0.05, Figure 5(c)). When ingredients were SM, the abundance of Fusobacteriota was significantly lower in the 100% SM group (*P* < 0.05), whereas the abundance of Planctomycetota, Chloroflexi, and Cyanobacteria was significantly higher in the 100% SM group (*P* < 0.05). When ingredients were FSM, in the 100% FSM group, Fusobacteriota abundance was significantly lower (*P* < 0.05), while Actinobacteriota and Verrucomicrobiota abundance were significantly higher in the 100% FSM group (*P* < 0.05). There was no significant interaction effect of ingredients and replacement level on the levels of intestinal flora at the phylum level (*P* < 0.05).

As shown in Figure 5(b), the dominant bacteria in the tilapia intestinal microbiota were *Cetobacterium* and *Mycobacterium* at the genus level. Ingredients or replacement levels significantly affected the abundance of *Cetobacterium* and *Plesiomonas* (*P* < 0.05, Figure 5(d)). *Cetobacterium* and *Plesiomonas* abundance was significantly lower in the 100% SM group when ingredients were SM (*P* < 0.05). When ingredients were FSM, *Mycobacterium* and *Bacillus* abundance was significantly higher in the 100% FSM group (*P* < 0.05), while *Cetobacterium* showed the opposite trend. There was no significant interaction effect of ingredients and replacement level on the levels of intestinal flora at the genus level (*P* > 0.05).

## 4. Discussion

SM has great potential for application in aquafeed production due to its high crude protein content and low price. In this study, the replacement of more than 40% of SBM with SM negatively affected the growth performance of tilapia. Similar results were reported by Shi et al. [8], where the substitution of more than 50% of SBM with SM reduced the growth of grass carp. Similarly, studies of gilthead sea bream have shown reduced growth performance when the dietary SM was above 24% [23]. The main factors limiting the utilization of SM include its incomplete dehulling, high crude fiber, and indigestible lignin content. These components increase the viscosity of the intestinal contents and delay gastric emptying, thereby reducing the utilization of dietary nutrients [10]. Further, large amounts of nonstarch polysaccharides are present in SM [24], which can reduce the nutrient utilization and growth performance of fish by binding to bile acids or obstructing the action of digestive enzymes and the movement of substrates in the intestine [25]. Furthermore, the low lysine content of SM is also a limiting factor [10]. In this study, despite the addition of lysine and methionine to the SM diet, other essential amino acids (EAAs) associated with fish growth may have contributed to the reduced growth. In contrast, the complete replacement of SBM with FSM had no significant effect on the growth performance of tilapia. These results indicate that the effects of SBM replacement with FSM are better than those achieved when replacing SBM with SM. Similarly, enzymolyzed SM can replace up to 75% more SBM in the grass carp diet compared to SM [8]. The main reason for this is the degradation of large proteins into small molecule peptides in FSM compared to SM (Figure S1). Small peptides are a class of small molecules that are hydrolyzed from proteins, easily digested and absorbed, and have multiple biological functions. They can increase the intake, feed efficiency, and body protein synthesis capacity of aquatic animals, thus promoting their growth [26]. With increases in the percentage of FSM substitution in the feed, there were no significant differences in the growth performance and FCR of tilapia. This may be attributed to the fact that FSM is rich in small peptides, which can reduce the antagonism between amino acids, avoid absorption competition during the absorption of free amino acids, and improve feed utilization by the animal [27].

The activities of digestive enzymes directly reflect the growth performance, digestive capacity, and nutritional status of aquatic animals, showcasing their vital role in nutrient digestion [28]. In the present study, amylase, lipase, and trypsin activities showed a tendency to increase and then decrease with increasing levels of SM substitution, with amylase and lipase activities attaining their maximum values at 20%, while trypsin activity attained its maximum value at 40%. As the level of SM substitution increased, the digestive enzyme activity decreased, probably due to its high chlorogenic acid and crude fiber contents. Chlorogenic acid can inhibit the activities of amylase, trypsin, and lipase and reduce the availability of proteins [29]. Coarse fiber can lead to changes in intestinal morphology and structure (a decrease in the intestinal villi height). This shortening of the villi reduces the surface area for nutrient absorption, leading to a decrease in digestive enzyme activity [2]. FSM had no significant effects on amylase and lipase, while an FSM replacement level exceeding 20% significantly decreased trypsin activity. The metabolic process of probiotics produces organic acids and increases the acidity of fermented feed. Therefore, increased intestinal acidity may possibly explain the decreased trypsin activity observed in the current study [30].

Animal growth is closely related to the apparent digestibility of the nutrients in the feed [31]. In this experiment, dry matter, total energy, and nutrient apparent digestibility gradually decreased with increasing levels of SM substitution. The decreases in the dry matter and gross energy apparent digestibility could be attributed to the indigestibility of cellulose in plant proteins [32]. Due to their high levels of cellulose, plant-based raw materials may accelerate the movement of surimi through the gut, thereby reducing the digestibility of dry matter and gross energy [33]. Similarly, Zhou et al. [34] suggested that an imbalance in EAAs in raw materials and the retention of nutrients in plant fiber materials were equally responsible for the decrease in protein digestibility. Together, these findings may possibly explain the current findings when SBM was replaced with SM. On the contrary, the apparent digestibility of dry matter and nutrients tended to increase as the level of FSM substitution increased. This may be related to the increase in peptides, free amino acids, and small molecular proteins through fermentation, as well as the reduced crude fiber and other anti-nutritional factors (Figure S1). Hassaan et al. [6] found that the replacement of more than 50% of fish meal with yeast-fermented SM significantly reduced the apparent digestibility of dry matter, energy, crude protein, and crude lipids. The differences in those results compared to the current study may be related to differences in the fermentation process of the raw material.

Gut histology is widely used to assess the potential negative effects of diets on the gut [35]. The height of the mucosal folds determines the absorptive surface area of the intestine, and the thickness of the muscularis propria can reflect the intestinal peristaltic capacity [20, 36]. In this experiment, both fold height, and muscle thickness tended to increase and then decrease as the level of SM substitution increased, with fold height achieving a maximum at 20% and muscle thickness at 40%, although there was no significant difference between the substitution groups and the control group, high dietary SM may inhibit intestinal digestive function. Conversely, reported that the intestinal muscular thickness of grass carp increased with increasing dietary SM levels [37]. The difference in the findings of the current study may be due to the differing fish species. Interestingly, FSM substitution had no effect on the gut histology of tilapia, possibly because of its lower crude fiber and higher probiotics contents.

The intestinal mucosal barriers include mechanical, chemical, immune, and biological barriers. These are widely involved in maintaining intestinal health, and the integrity of these barriers is often used to assess intestinal health [38]. Toxins, antigens, and pathogens cannot enter the body because of the intestinal barrier [39] but allow nutrients to enter the body [40]. Usually, serum diamine oxidase activity and endothelin level are used to measure the degree of damage to the intestinal mucosa. In this study, as the level of substitution with SM or FSM increased, the serum diamine oxidase activity and serum endothelin content showed decreasing and then increasing trends (both were lowest at the 20% substitution level). This suggests that low percentages of SM and FSM substitution are beneficial for increasing intestinal mucosal integrity. In addition, tight junction proteins (such as *occludin*, *claudins*, and *zos*) are major components of the intestinal mucosal mechanical barrier [41], controlling the paracellular gaps between the intestinal epithelial cells and preventing the spread of bacteria and antigens [42]. In this experiment, the replacement of 20% of SBM with SM or FSM upregulated the *zo-1*, *claudin*, and *occludin* mRNA expression levels, suggesting that substitution of low percentages of SBM with SM or FSM promotes the expression of intestinal tight junction structural proteins to enhance intestinal mucosal barrier function.

The intestinal inflammatory response is closely related to intestinal health and is usually assessed by examining the expression levels of pro- and anti-inflammatory factors [21]. In this study, 20% SM substitution downregulated the expression of *tnf-α*, *il-1β*, *il-6*, and *il-8* and upregulated the expression of *tgf-β1* and *tgf-β2*. In contrast, SM substitution levels of about 20% produced the opposite effects, suggesting that SM substitution at low levels can alleviate intestinal inflammation while substitution at high levels will induce intestinal inflammation. On the other hand, FSM exhibited better intestinal inflammation alleviation effects, Significantly downregulating the expression of intestinal *tnf-α*, *il-1β*, and *il-8* mRNA expression levels. A similar study also reported that a high percentage of replacement of SBM with SM caused an intestinal inflammatory response in grass carp, which was alleviated after enzymolysis [8].

The intestinal flora is an intricate symbiotic system closely related to host health, playing a role in physiological, nutritional, immune, and metabolic functions and dynamic homeostasis [43]. The diversity of the intestinal flora is an important indicator of the gut health of fish [44]. In this study, 100% substitution with SM or FSM significantly increased the diversity and richness of the intestinal flora of tilapia. Specifically, the Shannon, Chao1, and Ace indices in the FSM group were higher than those in the SM group. This may be related to the richness of small peptides, probiotics, organic acids, and flavonoids in FSM, which provide a more nutritious environment for the growth and reproduction of different flora. The findings of this study indicated that the replacement of SBM with SM or FSM increased the abundance and diversity of the intestinal flora. Conversely, Cao [37] found that SM supplementation had no significant effect on the intestinal *α*-diversity of grass carp, while enzymolyzed SM supplementation significantly increased the diversity of the intestinal flora. The variation in the findings of the above study as compared to the current study may be related to factors such as culture environment, feed composition, stage of growth and development, and fish species.

At the phylum level, the dominant bacteria in all groups were Fusobacteriota, Bacteroidota, Actinobacteriota, Proteobacteria, and Firmicutes. This is highly consistent with previous studies of this species [45, 46]. Fusobacteria are Gram-negative conditionally pathogenic bacteria that play a role in lipid utilization, nutrient digestion, and absorption, and disease prevalence of the host [47]. In this study, both the 100% SM and 100% FSM groups had significantly lower Fusobacteriota abundances than the control group, while the 100% FSM group had much lower Fusobacteriota abundances than the 100% SM group. This suggests that FSM is more favorable for nutrient digestion and absorption compared to SM as a substitution for SBM. Actinobacteria are capable of producing a wide range of beneficial metabolites, antibiotics, and bioactivities [48] and play a key role in intestinal permeability regulation, immunomodulation, and metabolism [49]. A substantial increase in Actinobacteria results in a greater abundance of beneficial substances [19] and favors intestinal homeostasis and immunomodulation [47]. In this study, 100% FSM substitution significantly increased the abundance of Actinobacteriota compared to the control and 100% SM substitution groups, suggesting that FSM is more beneficial for maintaining flora homeostasis and gut health. Planctomycetes break down heteropolysaccharides into short-chain fatty acids and polysaccharides, serving as a source of energy for the growth of shrimp [50]. In this study, the abundance of Planctomycetota was higher in the 100% SM and 100% FSM groups than in the 0% and 20% groups. Replacement of SBM with either SM or FSM provides energy for fish growth. Cyanobacteria can produce a variety of toxins (cyclic lipopeptides and microcystins), leading to damage to digestive organs [51]; however, their presence in small quantities contributes to fish growth [52]. Chloroflexi are parthenogenetic anaerobic bacteria that can photosynthesize and have a heterotrophic assimilation function. The presence of these bacteria in the gut of tilapia suggests that these bacteria enter the gut of tilapia as a colony from the aquatic environment. Verrucomicrobia can degrade excess mucins produced by the human intestinal lining [53]. In this study, the abundance of Cyanobacteria, green algae, and algal microbiota was higher in the 100% SM and 100% FSM groups than in the control group. The abundance of Cyanobacteria, Chloroflexi, and Verrucomicrobiota was higher in the 100% FSM group than in the 100% SM group, suggesting that a high proportion of FSM replacing SBM may damage intestinal digestive organs.

Further studies at the genus level showed that the abundance of *Cetobacterium* was significantly lower in the 100% SM and 100% FSM groups than in the 0% group. *Cetobacterium* is involved in host vitamin metabolism, produces vitamin B12, and antimicrobial peptides and can ferment peptide carbohydrates [54]. It has been suggested that a decrease in *Cetobacterium* abundance increases the body's immunity [47]. However, a comprehensive study is needed to analyze the specific regulatory mechanisms underlying the effects on the host. *Mycobacterium* avium is a chronic pathogen that causes *mycobacteriosis* in fish [55, 56, 57]. *Mycobacteriosis* is characterized by the presence of numerous granulomas of varying sizes in fish tissues. The target organs include the spleen, kidneys, and liver. Affected fish usually exhibit symptoms such as weight loss (anorexia), black pigmentation, and occasionally, spinal deformities and exophthalmos [55, 58]. In this study, the abundance of *Mycobacterium* was significantly higher in the 100% FSM group than in the control and 100% SM groups. It is worth noting that the fish did not show any signs of disease during culture. The specific mechanism underlying this finding requires further study. *Plesiomonas* is commonly found in the digestive tract of fish, aquatic animals, and various mammals. It is extremely harmful to fish and other aquatic animals when it develops [59]. In this study, there were no significant differences between each of the SM and FSM substitution groups and the control group. *Bacillus* is a typical probiotic, and increases in the abundance of the *Bacillus* population can improve the antioxidant and immune ability of tilapia. In this study, the abundance of *Bacillus* was higher in both the 100% SM and 100% FSM groups than in the control group. The abundance of *Bacillus* was higher in the 100% FSM group than in the 100% SM group. This indicates that the replacement of SBM with either SM or FSM can improve the antioxidant and immunity activities of tilapia. However, FSM was found to be superior to SM. Taken together, the above results indicate that replacing SBM with either SM or FSM does not cause gut flora dysbiosis in tilapia. This may be due to the high content of chlorogenic acid in both SM and FSM, which plays a role in maintaining the intestinal microbial balance [60].

## 5. Conclusions

In summary, our data confirm that replacing more than 40% of SBM with SM induces gut inflammation, damages gut health, and decreases growth performance, whereas FSM replacement of SBM did not negatively affect tilapia growth and health, it also did not have a significant ameliorative effect, with some parameters negatively affected at high replacement levels. Therefore, FSM replacement of SBM levels above 80% is not recommended.

## Figures and Tables

**Table 1:** Nutritional composition (%, dry matter basis) of sunflower meal (SM) and fermented sunflower meal (FSM).

	SM	FSM
Crude protein	40.00	40.78
Crude lipid	1.00	0.40
Aspartic acid	3.64	3.54
Threonine	1.50	1.50
Serine	1.73	1.67
Glutamic acid	8.24	7.95
Glycine	2.42	2.36
Alanine	1.79	1.88
Cystine	0.47	0.49
Valine	1.96	1.91
Methionine	0.54	0.65
Isoleucine	1.56	1.58
Leucine	2.61	2.50
Tyrosine	0.87	0.83
Phenylalanine	1.90	1.83
Lysine	1.45	1.50
Histidine	1.10	0.99
Arginine	3.25	3.06
Proline	1.68	1.78
Total amino acid	36.71	36.02

Tryptophan was not detected due to acid hydrolysis.

**Table 2:** Ingredients and proximate composition of the experimental diets (%, dry matter basis).

Ingredients	Control	Replacement level of SBM with SM					Replacement level of SBM with FSM				
		20%	40%	60%	80%	100%	20%	40%	60%	80%	100%
Fish meal	3.00	3.00	3.00	3.00	3.00	3.00	3.00	3.00	3.00	3.00	3.00
Chicken meal	2.00	2.00	2.00	2.00	2.00	2.00	2.00	2.00	2.00	2.00	2.00
Rapeseed meal	10.00	10.00	10.00	10.00	10.00	10.00	10.00	10.00	10.00	10.00	10.00
Soybean meal (SBM)	25.00	20.00	15.00	10.00	5.00	0.00	20.00	15.00	10.00	5.00	0.00
Sunflower meal (SM)	0.00	7.50	15.00	22.50	30.00	37.50	—	—	—	—	—
Fermented sunflower meal (FSM)	—	—	—	—	—	—	7.50	15.00	22.50	30.00	37.50
Wheat flour	20.00	20.00	20.00	20.00	20.00	20.00	20.00	20.00	20.00	20.00	20.00
Wheat bran	30.89	28.25	25.61	22.97	20.33	17.69	28.20	25.51	22.82	20.14	17.45
Soybean oil	4.70	4.76	4.82	4.88	4.94	5.00	4.81	4.92	5.03	5.13	5.24
Soybean lecithin	0.50	0.50	0.50	0.50	0.50	0.50	0.50	0.50	0.50	0.50	0.50
L-lysine	0.11	0.19	0.28	0.36	0.45	0.53	0.19	0.28	0.36	0.45	0.53
DL-methionine	0.22	0.22	0.21	0.21	0.20	0.20	0.22	0.21	0.21	0.20	0.20
Additive premix^a^	1.20	1.20	1.20	1.20	1.20	1.20	1.20	1.20	1.20	1.20	1.20
Others^b^	2.38	2.38	2.38	2.38	2.38	2.38	2.38	2.38	2.38	2.38	2.38
Proximate composition											
Dry matter (DM, %)	91.17	91.23	91.49	91.08	91.07	90.92	90.79	90.85	90.80	90.67	90.70
Crude protein (% DM)	31.24	31.01	31.23	30.97	31.23	31.18	31.35	31.22	31.28	30.98	31.13
Crude lipid (% DM)	7.38	7.12	7.24	7.17	7.26	7.34	7.47	7.61	7.17	7.63	7.60
Ash (% DM)	7.57	7.55	7.63	7.73	7.70	7.72	7.37	7.34	7.72	7.86	7.98
Gross energy (kJ/g DM)	21.34	21.28	21.30	21.41	21.40	21.60	21.38	21.50	21.52	21.54	21.46

^a^Additive premixes (g/kg mixture): vitamin A, 0.20 g; vitamin D_3_, 0.003 g; vitamin E, 4.40 g; vitamin K_3_, 0.66 g; vitamin B_1_, 0.33 g; vitamin B_2_, 0.88 g; vitamin B_6_, 0.73 g; vitamin B_12_, 0.001 g; nicotinic acid, 2.89 g; calcium pantothenate, 1.64 g; folic acid, 0.07 g; biotin, 0.003 g; vitamin C, 10.01 g; FeSO_4_·7H_2_O, 52.87 g; H_3_ClCu_2_O_3_, 0.65 g; ZnSO_4_·7H_2_O, 43.15 g; MnSO_4_·7H_2_O, 31.56 g; MgSO_4_·H_2_O, 44.65 g; Ca (IO_3_)_2_, 0.42 g; Na_2_SeO_3_, 0.11 g; CoCl_2_·6H_2_O, 0.14 g. ^b^ Others included 1.80% Ca (H_2_PO_4_)_2_, 0.20% NaCl, 0.30% choline chloride, 0.03% vitamin C, 0.05% Y_2_O_3_.

**Table 3:** Primer sequence for real-time quantitative PCR.

Gene	Primer sequence (5′ - 3′)		Accession no.
*Tnf-α*	Forward	TAGAAGGCAGCGACTCAA	NM_001279533.1
	Reverse	CCTGGCTGTAGACGAAGT	
*Il-1β*	Forward	GACAGCCAAAAGAGGAGC	XM_019365844.2
	Reverse	TCTCAGCGATGGGTGTAG	
*Il-6*	Forward	ATAGCAAGCATCTACACGCATCTCC	XM_003453898.2
	Reverse	GGGCTGCCAGGGAATTGTAAGTC	
*Il-8*	Forward	GCACTGCCGCTGCATTAAG	NM_001279704.1
	Reverse	GCAGTGGGAGTTGGGAAGAA	
*Il-10*	Forward	CTGCTAGATCAGTCCGTCGAA	XM_013269189.3
	Reverse	GCAGAACCGTGTCCAGGTAA	
*Tgf-β1*	Forward	TGCGGCACCCAATCACACAAC	XM_025897821.1
	Reverse	GTTAGCATAGTAACCCGTTGGC	
*Tgf-β2*	Forward	GCTCACGATCTTCCGTCTTC	NM_001311314
	Reverse	CACTCCCCCTCTGTTTGTGT	
*Claudin*	Forward	GTCTGTTTCTGGGCGTGGTGTC	XM_019367708.2
	Reverse	ACTCCGACTGACTCCTCATCTTCC	
*Occludin*	Forward	GGAGGAAAGCCGCAGTGTTCAG	XM_025899615.1
	Reverse	GTCGTAGGCATCGTCATTGTAGGAG	
*Zo-1*	Forward	ACATCGTGCGCTCCAACCAT	XM_019358174
	Reverse	GGCTGGACTGTGCTTGTGGT	
*β-Actin*	Forward	CCACACAGTGCCCATCTACGA	XM_003443127.5
	Reverse	CCACGCTCTGTCAGGATCTTCA	

*Tnf-α*: tumor necrosis factor-alpha; *Il-1β*: interleukin-1 beta; *Il-6*: interleukin-6; *Il-8*: interleukin-8; *Il-10*: interleukin-10; *Tgf-β1*: transforming growth factor beta 1; *Tgf-β2*: transforming growth factor beta 2; *Zo-1*: zonula occludens protein-1.

**Table 4:** Effect of replacing soybean meal (SBM) with sunflower meal (SM) or fermented sunflower meal (FSM) on growth performance and feed utilization of tilapia.

Dietary treatments		FBW (g)	WG	FI (%)	SGR (%/day)	FCR	PER	SR (%)
Ingredients	Replacement levels							
SM	0%	80.34 ± 1.40^ab^	11.25 ± 0.23^ab^	2.81 ± 0.07^c^	3.98 ± 0.03^ab^	1.04 ± 0.03^c^	3.08 ± 0.08^a^	98.09 ± 0.95^a^
	20%	81.24 ± 1.03^a^	11.40 ± 0.16^a^	2.92 ± 0.03^bc^	4.00 ± 0.02^a^	1.08 ± 0.01^bc^	2.98 ± 0.02^ab^	93.33 ± 1.90^b^
	40%	77.64 ± 0.82^bc^	10.84 ± 0.13^bc^	2.94 ± 0.03^ab^	3.92 ± 0.02^bc^	1.09 ± 0.01^b^	2.93 ± 0.03^bc^	97.14 ± 0.00^ab^
	60%	76.89 ± 0.51^c^	10.75 ± 0.09^c^	2.89 ± 0.02^bc^	3.91 ± 0.01^c^	1.08 ± 0.01^bc^	2.99 ± 0.02^ab^	99.05 ± 0.95^a^
	80%	76.46 ± 0.48^c^	10.68 ± 0.07^c^	2.94 ± 0.04^ab^	3.90 ± 0.01^c^	1.10 ± 0.02^ab^	2.92 ± 0.04^bc^	98.10 ± 1.90^a^
	100%	74.82 ± 1.09^c^	10.43 ± 0.17^c^	3.05 ± 0.02^a^	3.87 ± 0.02^c^	1.14 ± 0.01^a^	2.80 ± 0.02^c^	96.19 ± 0.95^ab^

FSM	0%	80.34 ± 1.40	11.25 ± 0.23	2.81 ± 0.07	3.98 ± 0.03	1.04 ± 0.03	3.08 ± 0.08	98.09 ± 0.95
	20%	82.46 ± 2.49	11.58 ± 0.38	2.79 ± 0.00	4.02 ± 0.05	1.03 ± 0.01	3.10 ± 0.02	96.19 ± 2.52
	40%	81.50 ± 0.77	11.42 ± 0.12	2.76 ± 0.02	4.00 ± 0.02	1.02 ± 0.01	3.14 ± 0.03	98.09 ± 0.95
	60%	80.08 ± 0.28	11.21 ± 0.04	2.75 ± 0.01	3.97 ± 0.01	1.02 ± 0.00	3.13 ± 0.01	100.00 ± 0.00
	80%	79.68 ± 0.87	11.16 ± 0.14	2.81 ± 0.03	3.97 ± 0.02	1.04 ± 0.01	3.09 ± 0.03	98.10 ± 1.90
	100%	78.42 ± 0.39	10.98 ± 0.07	2.80 ± 0.01	3.94 ± 0.01	1.04 ± 0.01	3.06 ± 0.02	100.00 ± 0.00

Ingredients								
SM		77.90	10.89	2.92	3.93	1.09	2.95	96.98
FSM		80.41^†^	11.27^†^	2.79^†^	3.98^†^	1.03^†^	3.10^†^	98.41
Replacement levels								
0%		80.34^ab^	11.25^ab^	2.81	3.98^ab^	1.04	3.08	98.09^a^
20%		81.85^a^	11.49^a^	2.86	4.01^a^	1.06	3.04	94.76^b^
40%		79.57^abc^	11.13^abc^	2.85	3.96^abc^	1.06	3.04	97.62^a^
60%		78.49^bc^	10.98^bc^	2.82	3.94^bc^	1.05	3.06	99.53^a^
80%		78.07^bc^	10.92^bc^	2.88	3.94^bc^	1.07	3.01	98.10^a^
100%		76.62^c^	10.71^c^	2.93	3.91^c^	1.09^a^	2.93	98.10^a^
Two-way ANOVA								
Ingredient		0.001	0.001	<0.001	0.001	<0.001	<0.001	0.078
Replacement level		0.002	0.003	0.042	0.002	0.016	0.021	0.043
Ingredient × replacement level		0.470	0.514	0.041	0.488	0.031	0.077	0.646

Data are expressed as mean ± SEM (*n* = 3). ^a,b,c^ Means with different superscript letters in the same column are significantly different (*P*  < 0.05) among treatments with SM or replacement levels. ^†^*P*  < 0.05, SM versus FSM. Weight gain (WG) = (final body weight − initial body weight)/initial body weight; feed intake (FI, %) = feed consumption/((initial body weight + final body weight)/2)/feeding days × 100; special growth rate (SGR, %/day) = (ln (final body weight) − ln (initial body weight))/feeding days × 100; feed conversion ratio (FCR) = dry weight of ingested feed/(final body weight − initial body weight); protein efficiency ratio (PER) = (final average weight − initial average weight)/(feed intake per fish × crude protein content of feed); survival rate (SR, %) = final fish number/initial fish number × 100.

**Table 5:** Effect of replacing soybean meal (SBM) with sunflower meal (SM) and fermented sunflower meal (FSM) on digestive enzyme activity of tilapia.

Dietary treatments		Amylase (U/mg protein)	Lipase (U/g protein)	Trypsin (U/mg protein)
Ingredients	Replacement levels			
SM	0%	27.83 ± 1.82^abc^	1.12 ± 0.02^bc^	408.83 ± 43.75^ab^
	20%	30.16 ± 2.34^a^	1.51 ± 0.23^a^	424.31 ± 28.99^ab^
	40%	29.57 ± 2.51^ab^	1.26 ± 0.08^ab^	432.17 ± 17.32^a^
	60%	22.92 ± 2.35^cd^	0.98 ± 0.10^bc^	309.04 ± 26.42^b^
	80%	23.15 ± 1.59^bcd^	0.83 ± 0.03^c^	369.32 ± 55.43^ab^
	100%	20.95 ± 0.93^d^	0.84 ± 0.07^c^	376.33 ± 27.62^ab^

FSM	0%	27.83 ± 1.82	1.12 ± 0.02	408.83 ± 43.75^x^
	20%	30.73 ± 1.94	1.14 ± 0.06	441.92 ± 26.13^x^
	40%	27.58 ± 1.01	1.15 ± 0.14	300.07 ± 23.10^y^
	60%	27.74 ± 0.61	1.12 ± 0.23	291.33 ± 11.92^y^
	80%	27.53 ± 0.85	1.09 ± 0.05	306.69 ± 0.79^y^
	100%	27.21 ± 1.00	0.80 ± 0.02	316.47 ± 24.56^y^

Ingredients				
SM	—	25.76	1.09	386.67
FSM	—	28.10	1.07	344.22
Replacement levels				
0%	—	27.83^abc^	1.12^ab^	408.83^ab^
20%	—	30.45^a^	1.33^a^	433.12^a^
40%	—	28.58^ab^	1.21^ab^	366.12^abc^
60%	—	25.33^bc^	1.05^bc^	300.19^c^
80%	—	25.34^bc^	0.96^bc^	338.01^bc^
100%	—	24.08^c^	0.82^c^	346.40^bc^
Two-way ANOVA				
Ingredient		0.025	0.769	0.026
Replacement level		0.008	0.002	0.003
Ingredient × replacement level		0.140	0.146	0.231

Data are expressed as mean ± SEM (*n* = 3). ^a,b,c,d^ Means with different superscript letters in the same column are significantly different (*P*  < 0.05) among treatments with SM or replacement levels; ^x,y^ Means with different superscript letters in the same column are significantly different (*P*  < 0.05) among treatments with FSM.

**Table 6:** Effect of replacing soybean meal (SBM) with sunflower meal (SM) and fermented sunflower meal (FSM) on nutrient apparent digestibility of tilapia.

Dietary treatments		Dry matter (%)	Energy (%)	Crude protein (%)	Crude lipid (%)	Crude ash (%)
Ingredients	Replacement levels					
SM	0%	73.09 ± 0.16^a^	85.08 ± 0.01^a^	85.71 ± 0.24^a^	89.16 ± 0.91^ab^	50.40 ± 2.55^a^
	20%	70.76 ± 0.19^b^	83.70 ± 0.01^b^	85.61 ± 0.66^a^	90.39 ± 0.73^a^	47.39 ± 2.76^a^
	40%	69.94 ± 0.13^c^	83.45 ± 0.01^c^	83.91 ± 0.35^b^	89.34 ± 1.16^ab^	47.47 ± 0.52^a^
	60%	68.75 ± 0.29^d^	82.51 ± 0.01^d^	83.45 ± 0.70^b^	87.91 ± 0.73^abc^	45.12 ± 1.23^a^
	80%	67.81 ± 0.23^e^	81.79 ± 0.02^e^	83.72 ± 0.23^b^	86.24 ± 1.83^bc^	45.39 ± 0.34^a^
	100%	64.85 ± 0.00^f^	80.17 ± 0.01^f^	83.59 ± 0.50^b^	85.05 ± 1.12^c^	39.81 ± 0.47^b^

FSM	0%	73.09 ± 0.16^z^	85.08 ± 0.01^z^	85.71 ± 0.24	89.16 ± 0.91^y^	50.40 ± 2.55^y^
	20%	75.44 ± 0.14^wx^	86.28 ± 0.01^v^	87.19 ± 0.41	90.29 ± 0.48^xy^	47.16 ± 0.41^y^
	40%	74.60 ± 0.00^y^	86.02 ± 0.01^w^	87.22 ± 1.75	91.69 ± 0.37^x^	46.90 ± 1.44^y^
	60%	75.13 ± 0.05^xy^	85.96 ± 0.01^x^	87.64 ± 0.45	92.10 ± 0.62^x^	51.40 ± 1.84^y^
	80%	75.23 ± 0.27^wx^	85.88 ± 0.01^y^	87.02 ± 0.45	92.05 ± 0.34^x^	56.50 ± 0.39^x^
	100%	75.79 ± 0.30^w^	86.26 ± 0.00^v^	87.37 ± 0.16	91.56 ± 0.39^x^	57.56 ± 0.47^x^

Ingredients						
SM	—	69.20	82.79	84.33	88.01	45.93
FSM	—	74.88^†^	85.91^†^	87.02^†^	91.14^†^	51.65^†^
Replacement levels						
0%	—	73.09	85.08	85.71	89.16	50.40
20%	—	73.10	84.99	86.40	90.34	47.28
40%	—	72.27	84.74	85.57	90.51	47.19
60%	—	71.94	84.24	85.54	90.01	48.26
80%	—	71.52	83.84	85.37	89.15	50.95
100%	—	70.32	83.22	85.48	88.31	48.69
Two-way ANOVA						
Ingredient		<0.001	<0.001	<0.001	<0.001	<0.001
Replacement level		<0.001	<0.001	0.669	0.153	0.104
Ingredient × replacement level		<0.001	<0.001	0.032	0.003	<0.001

Data are expressed as mean ± SEM (*n* = 3). ^a,b,c,d,e,f^ Means with different superscript letters in the same column are significantly different (*P*  < 0.05) among treatments with SM or replacement levels; ^w,x,y,z^ Means with different superscript letters in the same column are significantly different (*P*  < 0.05) among treatments with FSM. ^†^*P*  < 0.05, SM versus FSM. Apparent digestibility coefficient of dry matter (%) = 100 × (1 – (dietary Y_2_O_3_ level/feces Y_2_O_3_ level)); apparent digestibility coefficient of nutrients (%) = 100 × (1– (dietary Y_2_O_3_ level/feces Y_2_O_3_ level) × (feces nutrient level/dietary nutrient level)).

**Table 7:** Effect of replacing soybean meal (SBM) with sunflower meal (SM) and fermented sunflower meal (FSM) on intestinal permeability of tilapia.

Dietary treatments		Diamine oxidase (U/mL)	Endothelin (ng/mL)
Ingredients	Replacement levels		
SM	0%	23.27 ± 0.50^a^	55.23 ± 0.51^b^
	20%	9.95 ± 0.84^d^	52.46 ± 0.62^b^
	40%	14.62 ± 0.33^c^	54.12 ± 1.23^b^
	60%	9.53 ± 0.79^d^	56.34 ± 0.29^b^
	80%	13.39 ± 0.34^c^	56.01 ± 0.67^b^
	100%	21.18 ± 0.34^b^	63.11 ± 4.41^a^

FSM	0%	23.27 ± 0.50^x^	55.23 ± 0.51^x^
	20%	12.82 ± 0.59^y^	49.90 ± 1.02^y^
	40%	20.94 ± 0.76^x^	50.24 ± 0.33^y^
	60%	19.17 ± 2.75^x^	50.68 ± 1.92^y^
	80%	14.56 ± 0.09^y^	50.35 ± 0.29^y^
	100%	21.72 ± 0.87^x^	50.90 ± 1.20^y^

Ingredients			
SM	—	15.32	56.21
FSM	—	18.75^†^	51.22^†^
Replacement levels			
0%	—	23.27^a^	55.23^ab^
20%	—	11.39^c^	51.18^b^
40%	—	17.78^b^	52.18^ab^
60%	—	14.35^bc^	53.51^ab^
80%	—	13.98^c^	53.18^ab^
100%	—	21.45^a^	57.01^a^
Two-way ANOVA			
Ingredient		<0.001	<0.001
Replacement level		<0.001	0.013
Ingredient × replacement level		<0.001	0.015

Data are expressed as mean ± SEM (*n* = 3). ^a,b,c,d^ Means with different superscript letters in the same column are significantly different (*P*  < 0.05) among treatments with SM or replacement levels; ^x,y^ Means with different superscript letters in the same column are significantly different (*P*  < 0.05) among treatments with FSM. ^†^*P*  < 0.05, SM versus FSM.

**Table 8:** Effect of replacing soybean meal (SBM) with sunflower meal (SM) and fermented sunflower meal (FSM) on intestinal histomorphology of tilapia.

Dietary treatments		Fold height (*µ*m)	Muscular thickness (*µ*m)
Ingredients	Replacement levels		
SM	0%	319.20 ± 32.27^ab^	45.12 ± 0.91^ab^
	20%	327.68 ± 15.81^a^	47.44 ± 3.36^a^
	40%	331.00 ± 10.24^a^	43.43 ± 2.52^abc^
	60%	274.11 ± 4.12^ab^	40.24 ± 1.79^bcd^
	80%	257.48 ± 15.33^b^	37.44 ± 1.79^cd^
	100%	286.67 ± 29.67^ab^	36.11 ± 1.37^d^

FSM	0%	319.20 ± 32.27^xy^	45.12 ± 0.91
	20%	250.10 ± 7.30^y^	39.97 ± 2.23
	40%	302.43 ± 5.62^xy^	40.88 ± 2.78
	60%	321.36 ± 24.67^xy^	41.58 ± 2.36
	80%	368.10 ± 8.46^x^	42.12 ± 1.36
	100%	355.34 ± 36.47^x^	42.44 ± 0.92

Ingredients			
SM	—	299.36	41.63
FSM	—	319.42	42.02
Replacement levels			
0%	—	319.20	45.12^a^
20%	—	288.89	43.71^ab^
40%	—	316.72	42.16^ab^
60%	—	297.74	40.91^ab^
80%	—	312.79	39.78^b^
100%	—	321.01	39.28^b^
Two-way ANOVA			
Ingredient		0.123	0.741
Replacement level		0.613	0.053
Ingredient × replacement level		0.003	0.027

Data are expressed as mean SEM (*n* = 3). ^a,b,c,d^ Means with different superscript letters in the same column are significantly different (*P*  < 0.05) among treatments with SM or replacement levels; ^x,y^ Means with different superscript letters in the same column are significantly different (*P*  < 0.05) among treatments with FSM.

**Table 9:** Effect of replacing soybean meal (SBM) with sunflower meal (SM) and fermented sunflower meal (FSM) on alpha-diversity index of tilapia intestinal flora.

Dietary treatments		Shannon	Simpson	Chao1	Ace	Goods coverage
Ingredients	Replacement levels					
SM	0%	2.43 ± 0.08^b^	0.69 ± 0.02^b^	361.95 ± 16.36^b^	373.35 ± 17.62^b^	1.00
	20%	2.57 ± 0.27^ab^	0.69 ± 0.03^b^	394.81 ± 25.03^ab^	406.47 ± 27.22^ab^	1.00
	100%	3.14 ± 0.17^a^	0.80 ± 0.02^a^	449.13 ± 18.65^a^	455.26 ± 25.95^a^	1.00

FSM	0%	2.43 ± 0.08^y^	0.69 ± 0.02	361.95 ± 16.36^y^	373.35 ± 17.62^y^	1.00
	20%	2.89 ± 0.30^xy^	0.69 ± 0.04	454.80 ± 55.23^xy^	471.84 ± 54.84^xy^	1.00
	100%	3.50 ± 0.18^x^	0.76 ± 0.03	510.97 ± 28.94^x^	516.35 ± 31.43^x^	1.00

Ingredients						
SM	—	2.71	0.73	401.96	411.69	1.00
FSM	—	2.94	0.71	442.57	453.85	1.00
Replacement levels						
0%	—	2.43^b^	0.69^b^	361.95^b^	373.35^b^	1.00
20%	—	2.73^b^	0.69^b^	424.81^ab^	439.16^ab^	1.00
100%	—	3.32^a^	0.78^a^	480.05^a^	485.81^a^	1.00
Two-way ANOVA						
Ingredient		0.180	0.521	0.115	0.121	0.234
Replacement level		0.001	0.009	0.004	0.008	0.641
Ingredient × replacement level		0.621	0.729	0.515	0.526	0.561

Data are expressed as mean ± SEM (*n* = 3). ^a,b^ Means with different superscript letters in the same column are significantly different (*P*  < 0.05) among treatments with SM or replacement levels; ^x,y^ Means with different superscript letters in the same column are significantly different (*P*  < 0.05) among treatments with FSM.

## Data Availability

The data used to support the findings of this study are available from the corresponding authors upon request.

## Supplementary Materials

Supplementary Materials Figure S1: the protein degradation of sunflower meal (SM) and fermented sunflower meal (FSM) by SDS–PAGE analysis.
