# Supplementary material for: Effects of Replacing Soybean Meal with Sunflower Meal or Fermented Sunflower Meal on the Growth Performance, Intestinal Microbiota, and Intestinal Health of Tilapia (GIFT, Oreochromis niloticus)
Source: Aquac Nutr. 2024 Jun 20;2024:9366952. doi: 10.1155/2024/9366952 (PMC11211014; doi:10.1155/2024/9366952)
Supplement: Supplementary Materials — Figure S1: the protein degradation of sunflower meal (SM) and fermented sunflower meal (FSM) by SDS–PAGE analysis. [file 9366952.f1.doc]

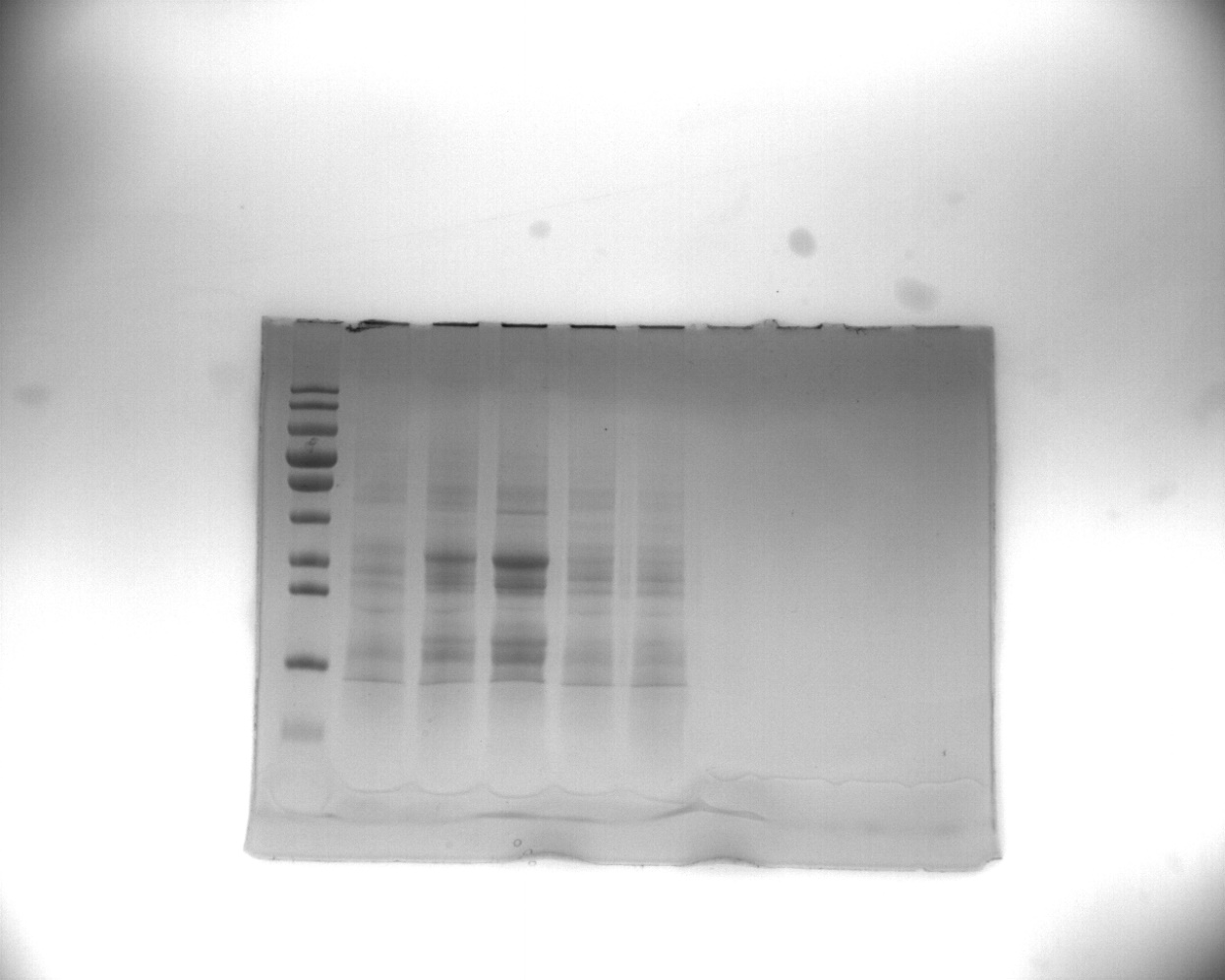


10

15

25

35

40

55

70

130

100

180

**1 2 3 4 5 6**

kDa

Figure 1: The protein degradation of sunflower meal (SM) and fermented sunflower meal (FSM) by SDS-PAGE analysis. 1, Maker; 2, Control; 3, 40% SM; 4, 100% SM; 5, 40% FSM; 6, 100% FSM.
